# A pathogenic non-coding RNA induces changes in dynamic DNA methylation of ribosomal RNA genes in host plants

**DOI:** 10.1093/nar/gkt968

**Published:** 2013-10-30

**Authors:** German Martinez, Mayte Castellano, Maria Tortosa, Vicente Pallas, Gustavo Gomez

**Affiliations:** Instituto de Biología Molecular y Celular de Plantas (IBMCP), Consejo Superior de Investigaciones Científicas (CSIC)-UPV, CPI, Edificio 8 E, Avenida de los Naranjos s/n, 46022 Valencia, Spain

## Abstract

Viroids are plant-pathogenic non-coding RNAs able to interfere with as yet poorly known host-regulatory pathways and to cause alterations recognized as diseases. The way in which these RNAs coerce the host to express symptoms remains to be totally deciphered. In recent years, diverse studies have proposed a close interplay between viroid-induced pathogenesis and RNA silencing, supporting the belief that viroid-derived small RNAs mediate the post-transcriptional cleavage of endogenous mRNAs by acting as elicitors of symptoms expression. Although the evidence supporting the role of viroid-derived small RNAs in pathogenesis is robust, the possibility that this phenomenon can be a more complex process, also involving viroid-induced alterations in plant gene expression at transcriptional levels, has been considered. Here we show that plants infected with the ‘Hop stunt viroid’ accumulate high levels of sRNAs derived from ribosomal transcripts. This effect was correlated with an increase in the transcription of ribosomal RNA (rRNA) precursors during infection. We observed that the transcriptional reactivation of rRNA genes correlates with a modification of DNA methylation in their promoter region and revealed that some rRNA genes are demethylated and transcriptionally reactivated during infection. This study reports a previously unknown mechanism associated with viroid (or any other pathogenic RNA) infection in plants providing new insights into aspects of host alterations induced by the viroid infectious cycle.

## INTRODUCTION

Impelled by their need to optimize a humble (250–400 nt) non-protein-coding genome to guarantee their infectious cycle, viroids have evolved into versatile molecular entities capable of interacting with the host-cell machinery at diverse functional levels (1). In some cases, this crosstalk can affect key host-regulatory pathways and cause phenotypic alterations recognized as plant diseases (2,3). Host factors and/or mechanisms associated with basic aspects of the life cycle of viroids, such as sub-cellular compartmentalization (4,5), replication (6–8) and movement (9–12), have been thoroughly studied in the past years to generate a relatively clear picture of the plant–viroid interaction (13). However, the way in which these tiny non-coding RNAs (ncRNAs) subvert the plant cell machinery by coercing the host to express symptoms remains a conundrum (3).

As viroids lack mRNA activity, it was initially assumed that viroid-induced pathogenesis must result from a direct interaction between their genome and host factors (proteins or nucleic acids). Based on this notion, early pathogenesis models envisioned the plant-symptom expression as a consequence of alterations in the endogenous RNA metabolism induced by base pair interactions between viroid and host ncRNAs, like U1 (14) or 7S (15). The identification of sequence/structural elements in the viroid genome (pathogenicity determinants) (16,17) supports the notion that these specific viroid domains may interact with yet-to-be-identified host factors interfering in their physiological role and consequently incite disease (1,3). In recent years, and enhanced by the advent of RNA silencing, diverse studies have provided evidence for the existence of close interplay between viroid-induced pathogenesis and this small RNA (sRNA)-dependent regulatory mechanism. The idea that viroid-derived sRNAs (vd-sRNAs) can mediate the post-transcriptional cleavage of endogenous mRNAs, acting as elicitors of symptoms expression (18–20), was reinforced by the observation that the expression of host symptoms is associated with post-transcriptional RNA silencing in representative members of both *Pospiviroidae* (21) and *Avsunviroidae* (22) families. Despite strong evidence supporting the role of vd-sRNAs as inductors of host symptoms, the possibility that this phenomenon can be a more complex process, also involving viroid-induced alterations in plant gene expression at transcriptional levels, cannot be ruled out (3,13).

Genome activity at the transcriptional level in plants is regulated by epigenetic modifications of DNA and histones. Cytosine DNA methylation in plants is a stable epigenetic mark, basic for the maintenance of genome stability, including regulation of coding and non-coding regions (23). Methylation takes place in three different sequence contexts, CG and CHG (symmetric) and CHH (asymmetric, where H refers to A, T or C) (24). *De novo* DNA methylation is regulated by DOMAINS REARRANGED METHYLTRANSFERASE 2 (DRM2), with three different pathways being involved in its maintenance. DNA METHYLTRANSFERASE 1 (MET1) and DNA CHROMOMETHYLASE3 (CMT3), respectively, maintain CG and CHG methylation through DNA replication, whereas CHH methylation requires *de novo* continuous maintenance by DRM2 (24,25). sRNAs are important in *de novo* establishment and reinforcement of stable DNA methylation because they can target homologous DNA sequences and induce cytosine DNA methylation in all three contexts. A specific RNA silencing pathway, termed RNA-directed DNA methylation (RdDM), mediates the biosynthesis of these epigenetically active sRNAs (26). In the *Arabidopsis–*RdDM pathway, single-strand transcripts originated by RNA polymerase IV (PolIV) are used by RNA-dependent RNA polymerase 2 (RDR2) as substrates to generate dsRNAs, which are subsequently processed by Dicer-like 3 (DCL3) into 23–24 nt siRNAs (25). These siRNAs guide *Argonaute* (AGO4/AGO6) proteins (likely through an siRNA-nascent PolV transcript base pairing mechanism) to target genomic regions (27). Next, DRM2 is recruited to direct both symmetric and asymmetric *de novo* methylation. Conversely, *Arabidopsis* encodes a family of DNA glycosylases/lyases that mediate the active demethylation of methylcytosines (28). Recently, an alternative RDR6 21/22-nt siRNA-dependent pathway has been described to mediate the initiation of *de novo* DNA methylation of transposable elements (29).

The first evidence linking viroid infection and transcriptional regulation in plants was provided by the demonstration that *Potato spindle tuber viroid* (PSTVd) replication directs *de novo* methylation of cognate DNA sequences in viroid-expressing transgenic tobacco plants (30). Furthermore, other studies have shown that diverse host genes exhibit a transcriptional alteration during PSTVd (31,32), *Citrus viroid* III (33), *Peach latent mosaic viroid* (34) and *Citrus exocortis viroid* (35) infections. Although all this experimental evidence may support the idea that viroid infection incites alterations in plant gene expression at transcriptional levels (13), viroid-induced methylation of the host gene(s) or disruption of methylation pathways (which may eventually result in symptom expression) has not yet been reported in natural infections (3).

To address this issue, we first analyzed the alteration in the levels of endogenous sRNAs, associated with ‘Hop stunt viroid’ (HSVd) infection in cucumber (*Cucumis sativus*) plants. Our results indicate that HSVd-infected plants differentially accumulate high levels of the sRNAs derived from ribosomal transcripts (rb-sRNAs) associated with an increasing accumulation of ribosomal RNA precursors(pre-rRNAs) during infection. Finally, we observed that deregulation in rRNA transcriptional activity correlates with a dynamic modification of the DNA methylation level during the infection process to provide unprecedented insight into the potential endogenous regulatory pathways affected during the viroid life cycle in infected plants.

## MATERIALS AND METHODS

### Plant material

Cucumber (*C. sativus* cv Suyo) plants were agroinoculated with *Agrobacterium tumefaciens* strain C58C1 transformed with a binary pMOG800 vector carrying a head-to-tail infectious dimeric HSVd cDNA (Y09352) (36), as previously described (21) (Supplementary Materials). Mock-inoculated cucumber plants were used as a negative control. Plants were maintained in growing chambers and were analyzed at 10 (T1), 20 (T2) and 30 (T3) days post-infiltration (dpi) (Supplementary Figure S4A).

### sRNA library information

The sRNA sequences used in this work were obtained from a library generated by starting from an sRNA population recovered from leaves and phloem exudates of healthy and HSVd-infected cucumber (*C. sativus*) plants and sequenced by 454 Life Science Technology (Lifesequencing, Branford, CT, USA; www.lifesequencing.com) [(37) - NCBI/SRA accession code SRP001408]. The vd-sRNAs, recovered from infected plants, were filtered out from this analysis.

### RNA isolation

Total RNA was extracted from the leaves (∼0.1 g) of different infected and healthy cucumber plants using the TRI reagent (SIGMA, St. Louis, MO, USA) according to the manufacturer’s instructions. The low-molecular-weight RNA (<200 nt) fraction was enriched from total RNA using MIRACLE (miRNA isolation Kit, STRATAGENE) according to the manufacturer’s instructions (Supplementary Materials).

### Northern blot assays

Total RNA was analyzed by electrophoresis under denaturing conditions in 1% agarose gels with 0.25× Tris-Borate-EDTA (TBE) and 8 M urea (38). The RNA was blotted on nylon membranes (ROCHE Diagnostics GmbH, Mannheim, Germany) and was hybridized as previously described (11). Approximately 25 μg of low-molecular-weight RNA was loaded onto 20% polyacrylamide gels with 0.25× TBE and 8 M urea. RNA was transferred to a nylon membrane (ROCHE Diagnostics GmbH, Mannheim, Germany). Hybridization was performed as previously described (39).

### Bisulfite conversion and sequencing

Total genomic DNA was extracted from the leaves (∼0.1 g) of different infected and healthy cucumber plants (Supplementary Figure S7A and B) (40). Bisulfite treatment was performed using the EpiTec Bisulfite kit (Qiagen) (Supplementary Material). Modified DNA was amplified by polymerase chain reaction (PCR) and was cloned (Supplementary Figure S7C). Between 13 and 24 clones were sequenced for each analyzed time in both the infected and mock-treated plants.

## RESULTS

### rRNA-derived sRNAs are highly recovered from HSVd-infected plants

**Figure 1.**
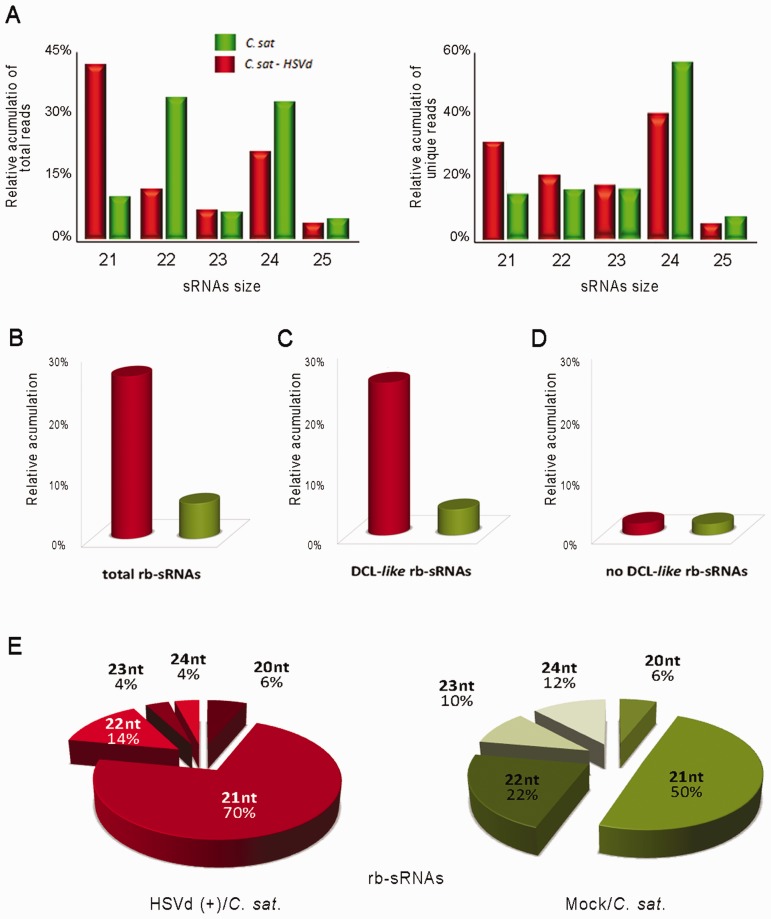
Characterization of the sRNAs recovered from cucumber leaves by deep sequencing. (**A**) Graphic representation of the differential accumulation and distribution of the total (left) and unique (right) reads of endogenous cucumber sRNAs ranging between 21 and 25 nt recovered from both the control and HSVd-infected samples analyzed at 30 days post-inoculation. (**B**) Differential recovering of rb-sRNAs from infected and healthy tissues. The accumulation of rb-sRNAs is expressed as the percentage of total rb-sRNAs from the overall sRNAs in the library. (**C**) and (**D**) show the relative accumulation of recovered rb-sRNAs with canonical (21–24 nt) and non-canonical (<21 and >24 nt) predicted sizes, respectively. (**E**) Graphic representation of the distribution of the total reads of rb-sRNAs (ranging between 21 and 25 nt) recovered from both the HSVd-infected (left) and control (right) analyzed samples.

To evaluate whether viroid infection induces alterations in the general profiles of host endogenous sRNAs, we compared the size distribution of the total reads recovered from HSVd-infected plants with the standard sRNA levels previously described for cucumber (41). As observed in Figure 1A (left), 21-nt sRNAs were the dominant size class in infected plants, whereas lengths of 22 and 24 nt were the most abundant in non-inoculated plants. The 23- and 25-nt-sized classes showed similar accumulation levels in both the control and HSVd-infected sRNAs populations. This scenario was in general reproduced (except for the 22-nt reads) when the unique sequences recovered from both datasets were analyzed (Figure 1A, right), indicating that HSVd infection is associated with changes in the endogenous sRNA population levels, being the 21-and 24-nt species up- and downregulated in infected plants, respectively.

**Figure 2.**
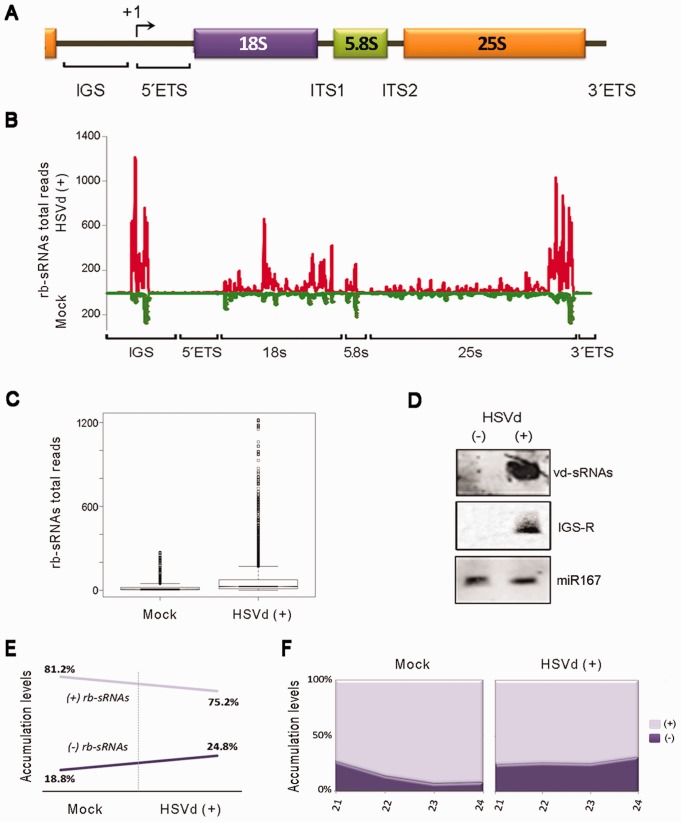
Analysis of ribosomal-derived sRNAs differentially expressed in infected cucumber plants. (**A**) Diagram (no scale) of the rRNA gene unit. The rRNA gene repeats are arranged in long tandem arrays of 45S rRNA genes, each including the region for the 18S, 5.8S and 25S rRNAs, and separated from adjacent units by an IGS. The transcription start site is indicated by +1. The ETS and ITS are removed during rRNA processing. (**B**) The rb-sRNAs recovered from the infected (above the X-axis) or the non-inoculated plants (below the X-axis) were plotted according to the position of their 5′-end onto the cucumber rRNA sequence. The values on the Y-axis represent the number of total reads in each library. The nucleotide positions −115 to +15 of the 45S rRNA-analyzed region are represented on the X-axis. (**C**) Comparison of the accumulation of the rb-sRNAs that map along the rRNA transcriptional unit between both the infected and control plants as shown in the box-plot (boxes represent the medians, and the first and third quartiles of the dataset; circles refer to the data whose values are beyond the quartiles. Comparison of the means of the boxes showed significant differences between both samples using a parametric *t*-test: arithmetic means for mock and infected plants are 9.49 and 37.66, respectively; *t* = 27.11; *P* < 2.2 × 10^−16^). (**D**) The hyper-accumulation in the infected samples of the rb-sRNAs derived from an ∼300-nt region of the IGS (IGS-R) was validated by northern blot assays. The miR167 equally recovered from both analyzed sRNA datasets was used as a load control. Hybridization with a probe against the HSVd-derived sRNAs (vd-sRNAs) was used as a positive control. (**E**) The relative accumulation of total rb-sRNAs complementary to rRNA transcripts increased in the HSVd-infected plants (left). A similar result was obtained when individually analyzing the expected canonical size of the rb-sRNAs (right).

When plant endogenous sRNAs recovered from infected and healthy plants were analyzed by pairwise alignment against the cucumber transcript database, we observed that the sRNAs derived from 45S ribosomal RNA were the most differentially accumulated population of sRNAs in viroid-infected plants (Figure 1B). The accumulation of 45S rRNA-derived sRNAs (rb-sRNAs) was at least 4-fold higher in infected (27.4%) compared with control (6.8%) plants. This difference slightly increased (24.2 and 4.9%, respectively) when considering only sRNAs with expected DCL canonical sizes ranging from 21 to 24 nt (Figure 1C). In contrast, negligible differences were found when considering the levels of the unexpected size (<21 to >24 nt in length) reads (Figure 1D). Consistent with the global sRNA population (Figure 1A), the rb-sRNAs of 21 and 24 nt were, respectively, up- and down recovered from the HSVd-infected dataset in comparison with control cucumber reads (Figure 1E). To examine the distribution of the rb-sRNA set, all the potentially DCL-processed sequences (21–24 nt) recovered from the healthy and HSVd-infected cucumber plants were mapped on the 45S rRNA transcriptional unit (Figure 2A). Despite both sRNA populations displaying a relatively heterogeneous distribution pattern along the rRNA sequence (Figure 2B), the total number of sequences matching throughout the rRNA gene significantly increased in infected plants as compared with the reads recovered from mock-inoculated cucumbers (Figure 2C; parametric *t*-test: arithmetic means of 37.6 and 9.4, for infected and mock-inoculated plants, respectively, *P* < 2.2 × 10^−^^16^). Moreover, we noticed two considerable differences between rb-sRNA profiles in both analyzed samples. First, in the infected plants, the differentially recovered rb-sRNAs are predominantly mapping to two homologous regions of 300 nt located in the intergenic spacer (IGS) region and the 3′-end of the 25s rRNA (Figure 2B). The northern blot analyses, using a probe corresponding to this specific IGS region, revealed a remarkable accumulation of rb-sRNAs in the infected plants (Figure 2D). Second, a major proportion of rb-sRNAs complementary to the rRNA transcripts from both polarities was recovered from the HSVd-infected plants (Figure 2E and F; Supplementary Figure S1), suggesting that the over-accumulation of rb-sRNAs observed in infected plants might originate from the rRNA-derived double-stranded RNAs processed in sRNAs.

Interestingly, the accumulation of sRNAs derived from 5S rRNA transcripts (5S sRNAs) was also increased in HSVd-infected (0.17%) compared with control (0.06%) plants (Supplementary Figure S2A). Consistent with observations in the 25S sRNA population, the 5S sRNAs of 21 and 24 nt were up- and downregulated in the HSVd-infected plants, respectively, and a major proportion of sRNAs complementary to 5S transcripts was recovered from the infected cucumber plants (Supplementary Figure S2B and C). When the 5S sRNAs were mapped onto their transcriptional unity, we observed that in the infected plants, the 5S sRNAs are heterogeneously distributed along the 5S rRNA sequence (Supplementary Figure S2D). However, the 5S sRNAs recovered from mock control disproportionately (72% of the analyzed reads) match to a region adjacent to the transcription start site. Importantly, the totality of these sRNAs was 24 nt long. All together, these results support the belief that in cucumber, HSVd infection is associated with a drastic alteration of rRNA-derived sRNA processing, mainly characterized by an increase in the accumulation of 21-nt-long sRNAs (potential products of the processing of ds-rRNAs) and a significant decrease of ribosomal-specific 24-nt sRNAs (assumed to be involved in maintenance of methylation status).

### rRNA precursors accumulate differentially during viroid infection

**Figure 3.**
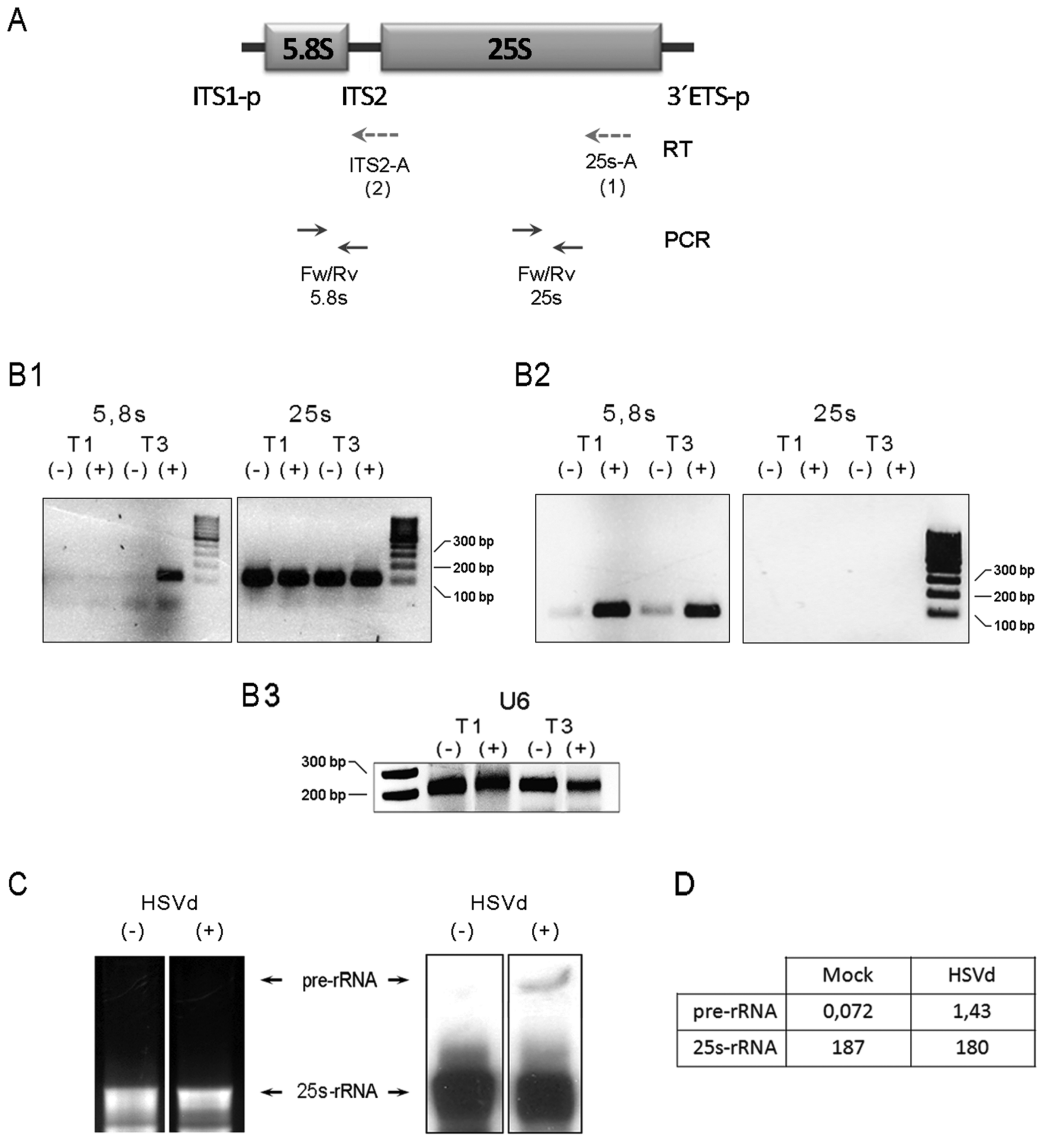
Precursor for rRNAs (pre-rRNAs) accumulates in infected plants. (**A**) Diagram (no scale) of the pre-27S rRNA (ITS1-p and 3′ETS-p refer to partially processed transcribed spacers). The dotted arrows below depict the oligos used for the RT reaction starting from the 25S 3′-end (25s-A/RT-1) and the ITS2 (ITS2-A/RT-2) regions of the pre-rRNA. Solid arrows indicate the oligos used in the PCR amplification. (**B**) The RT-PCR analyses of the pre-rRNA expression in the HSVd-infected (+) and mock-inoculated (−) plants at 10 (T1) and 30 (T3) days post-inoculation. The initial cDNAs were transcribed using the 25s-A (B1) and ITS2-A (B2) oligos, respectively. (*B*3) RT-PCR amplification of U6 Small nucleolar RNA (snoRNA) served as control for RNA load. (**C**) The accumulation of the pre-rRNA (marked with arrows) in the infected plants was validated by northern blot assays in the total RNAs extracted from plants at T3 using a probe complementary to the 3′-end of the 25S rRNA. (**D**) The band intensity was measured using the Image-J application http://www.imagej.en.softonic.com.

In *Arabidopsis*, the 45S rRNA genes (Figure 2A) are tandemly arrayed by hundreds at the chromosomal loci known as nucleolus organizers (42). RNA polymerase I (PolI) transcribes primary transcripts (called pre-rRNAs) that are then extensively processed into 18S, 5.8S and 25S rRNAs by the sequential deletion of external and internal transcribed spacers (ETS and ITS, respectively) (43). Each rRNA gene is separated from its adjacent one by an IGS, and its expression is regulated at several levels according to the physiological need for ribosomes, with one such level being the epigenetic on/off switch that controls the number of active rRNA genes (44). A deficiency in this regulatory mechanism, which controls rRNA transcriptional activity, has been recently associated with overproduction of rb-sRNAs in *Arabidopsis* mutants (45,46). To determine whether an alteration in the transcriptional activity of rRNA genes can be linked to the substantial accumulation of rb-sRNAs in HSVd-infected cucumber plants, we analyzed the accumulation of pre-rRNAs at specific points (T1: 10 dpi and T3: 30 dpi) during viroid infection. The reverse transcriptase-polymerase chain reaction (RT-PCR) assay was used to compare the pre-RNA levels, analyzing specifically two regions in the 27S rRNA transcription unit (Figure 3A). Two different primers complementary to the 3′-end of 25S rRNA (25s-A) and ITS2 (ITS2-A) were used to generate the cDNA template. The pair 5.8s-Fw/5.8s-Rv was used to differentially amplify by PCR the unprocessed rRNAs. Significant differences in the pre-rRNA transcript accumulation levels were found in viroid-infected plants in comparison with mock-inoculated controls at infection time T3 when using both the 25s-A and the ITS2-A primers for RT (Figure 3B, *B*1 and *B*2, respectively). In the analysis performed at T1 (10 dpi), we observed only a selective accumulation of pre-rRNAs when the PCR was done from the ITS2-A-generated cDNA. This slight incongruence, however, can be attributed to poorer efficiency in the generation of sufficiently long cDNA templates when using the 25 s-A primer, instead of ITS2-A, in the RT reaction. The differential accumulation of pre-rRNAs in infected plants at infection time T3 was corroborated by northern blot assays of 27 S pre-RNA levels (Figure 3C). The finding that HSVd infection is associated with an increase in the pre-rRNA levels suggests that the regulation of the transcriptional activity of rRNA genes might be impaired in viroid-infected cucumber.

### Viroid infection modifies dynamic rDNA methylation

**Figure 4.**
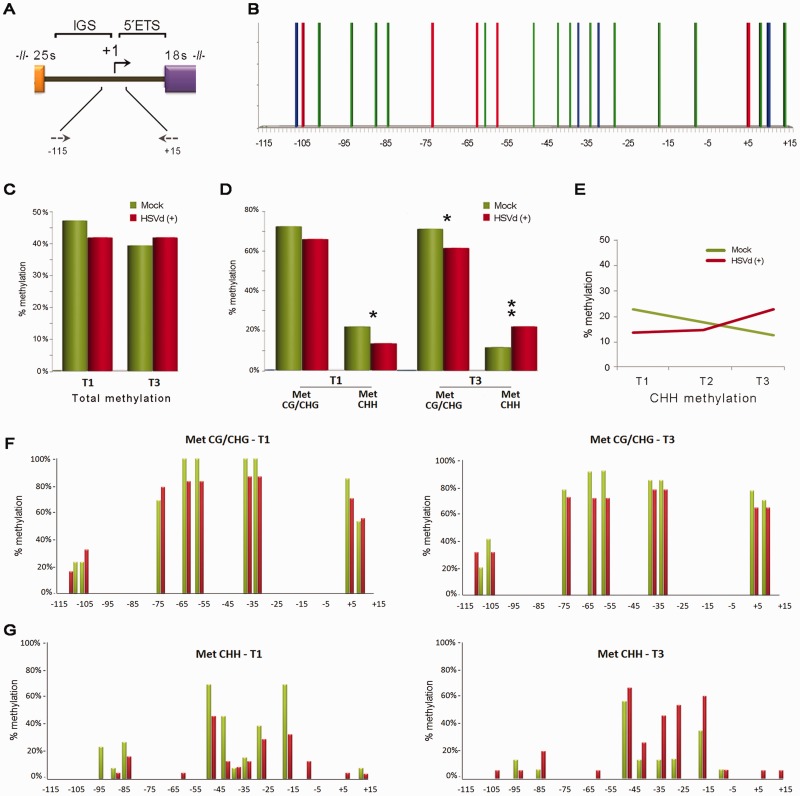
HSVd infection affects the methylation patterns in 45S rRNA genes. (**A**) Diagram of the rRNA gene intergenic region highlighting the promoter zone analyzed by bisulfite sequencing. The arrows represent the oligos used in the PCR assay and their relative position in the rRNA gene. (**B**) Graphic representation of the potential symmetric (CG–red bars and CHG–blue bars) and asymmetric (CHH–green bars) positions predicted to exist within the analyzed region. (**C**) Histogram documenting the relative (HSVd/mock) total DNA methylation levels at infection times T1 and T3. (**D**) Schematic representation of the differential analysis of both symmetric and asymmetric cytosine methylation at infection times T1 and T3 (paired *t*-test values T1: means symmetric methylation (mock) 0.73, (infected) 0.67, *t* = 1.604; means CHH methylation (mock) 0.22, (infected) 0.13, *t* = 2.180; T3: means symmetric methylation (mock) 0.72, (infected) 0.64, *t* = 2.481; means CHH methylation (mock) 0.11, (infected) 0.22, *t* = 3.047; *P < 0.05, **P < 0.01). (**E**) Evolution of the CHH methylation during HSVd infection in comparison with the level observed in the mock-inoculated plants. (**F**) Positions of methylcytosines in the analyzed regions displayed in the symmetric (CG and CHG) context. (**G**) Positions of methylcytosines in the analyzed regions displayed in the asymmetric context. The height of the bar represents the frequency at which cytosine was methylated at the analyzed infection times T1 (left) and T3 (right).

During diverse phases of the plants’ life cycle, rRNA genes are presumably in excess and are consequently silenced. At present, it is well accepted that the silencing of rRNA genes is a self-reinforcing regulatory phenomenon mediated by siRNA-directed cytosine methylation and heterochromatin formation (44,47). To investigate whether the transcriptional deregulation of rRNA genes (revealed as pre-rRNA accumulation) observed during viroid infection can be associated with reduced cytosine methylation, we analyzed by bisulfite sequencing, which identifies the position of methylated and unmethylated cytosines, a 131-bp region of genomic 45 S rDNA (Figure 4A). Within this sequence, there were 9 symmetric (5 CG, 4 CHG) and 14 asymmetric (CHH) potential methylation sites (Figure 4B).

The DNA extracted from leaves of HSVd-infected and mock-inoculated cucumber plants at 10 dpi (T1) and 30 dpi (T3) was treated with bisulfite reagent to convert unmethylated cytosines into uracil, followed by PCR to amplify a specific rDNA sequence of 131 nt comprised between positions −115 and +15. PCR products were cloned, and the sequences of 13–24 clones were compiled for each time point. Methylation analysis revealed that at T1 point, HSVd infection resulted in a decrease in the relative number of methylated cytosine residues (83%) when compared with control plants. However, comparable relative methylation levels between infected and healthy plants were observed at 30 dpi (T3) (Figure 4C). When dynamic methylation was differentially analyzed for both symmetric and asymmetric pathways, we saw two different patterns. Symmetric methylation (CG/CHG) slightly decreased during infection and displayed lowered relative methylation levels between 10 and 12% at T1 and T3, respectively (Figure 4D and F; Supplementary Figure S3). These results indicate that HSVd infection is linked to CG/CHG demethylation, which may contribute to the transcriptional activation of normally silenced rDNA units, and can consequently induce the accumulation of pre-rRNA during pathogenesis. Conversely, in the asymmetric context (CHH), loss of relative methylation in infected plants at T1 was more significant than in the symmetric pathway, and a drastic increase in the relative methylation pattern of rDNA was observed at T3 of the HSVd-infected plants (Figure 4D and G; Supplementary Figure S4). To provide a more accurate picture of the changes occurring in the CHH methylation during viroid infection, we next studied an intermediate point of the infective cycle, at 20 dpi (T2), by bisulfite sequencing (Supplementary Figure S3). As shown in Figure 4E, the level of asymmetric methylation progressively increased at the analyzed infection times, thus explaining the equilibrium in the total methylation level observed at point T3 when comparing the HSVd-infected and mock-inoculated plants (Figure 4C). The alteration in the methylation levels of infected plants at T3 infection time was validated by combined bisulfite and restriction analysis (Supplementary Figure S5). Taken together, these results indicate that in the analyzed rDNA regions, while CG and CHG sites maintain a constant hypomethylated status during infection, the CHH sites show a dual scenario, being hypomethylated at T1 and actively hypermethylated during HSVd infection development.

To obtain additional evidence supporting the influence of HSVd infection on host transcriptional activity, we also analyze by bisulfite sequencing a 149-bp region of 5 S rDNA (Supplementary Figure S6A and B), another well-established target transcriptionally regulated by DNA methylation in plants (48). Consistent with observations for 45S rRNA, sequence analysis revealed that HSVd infection is also associated with alterations in the methylation status of the 5S rDNA. Methylation analysis revealed that at initial states of the infectious cycle (T1), the methylation levels observed in the analyzed region of the 5 S rDNA were comparable for both analyzed samples (Supplementary Figure S6C). However, HSVd infection resulted in a significant reduction in the relative number of methylated cytosine residues in infected plants (53.6%) compared with mock-inoculated controls (62.4%) at the T3 analyzed infection time (Supplementary Figure S6D). The loss of relative methylation in infected plants at T3 was comparable for cytosine residues in both the symmetric and asymmetric sequence context (Supplementary Figure S6D, E and F). Collectively, our results provide unprecedented insights into alterations in the dynamics of the host DNA methylation status in viroid-induced pathogenesis.

## DISCUSSION

Increasing evidence supports the notion that the study of viroid–host interactions can shed light on the regulatory pathways directed by ncRNAs in plants (2,13). Within this framework, deciphering the molecular basis of the viroid pathogenesis processes emerges as a fundamental milestone to be fulfilled. Since the first viroid-induced disease was reported, diverse pathogenesis models have been proposed (3). The actual prevailing notion linking viroid pathogenesis and RNA silencing essentially considers the interference of vd-sRNAs in endogenous RNA metabolism at a post-transcriptional level (19,20). However, the possibility that the expression of plant symptoms could eventually be a consequence of specific alterations in host regulatory mechanisms at a transcriptional level has also been envisioned (3,13,31–35). Interestingly, the data obtained in this work reveal that viroid-induced pathogenesis is also associated with transcriptional host alterations.

The initial observation that HSVd-infected cucumber plants show an unexpected hyper-accumulation of rb-sRNAs prompted us to speculate about the possibility that, during infection, HSVd [as previously observed for a symptomatic variant of *Peach latent mosaic viroid* (49)] could interfere in a yet undetermined manner in the rRNA maturation process, and that the rb-sRNAs highly recovered from infected plants could be a product of the DCL-mediated degradation of aberrantly processed rRNAs. Nonetheless, the observation that mature forms of rRNAs, such as 25 S, accumulate at comparable levels in both healthy and infected plants (Figure 3B and C) was incongruent with this supposition. Consequently, we explored an alternative option that rb-sRNA accumulation in infected plants could be the result of the viroid-induced transcriptional deregulation of rRNAs, resembling that recently observed in *Arabidopsis* where rb-sRNAs overproduction has been linked to deficiencies in the on/off switch controlling the number of active rRNA genes (46). Increased accumulation of 27 S pre-rRNA in infected plants, in parallel to the maintenance of equivalent levels of processed rRNA forms (i.e. 25 S), strongly suggests that, during infection, HSVd may induce the upregulation of some of the normally inoperative rRNA transcriptional units.

Bisulfite sequencing clearly correlated HSVd infection with dynamic DNA methylation changes taking place in the analyzed regulatory region (−115/+15) of 45 S- rDNA. The symmetrical cytosine methylation progressively decreased during infection, whereas the asymmetric *de novo* cytosine methylation also decreased at the initial infection time but actively increased throughout infection development. Interestingly, it has been previously reported in *Arabidopsis* that changes in the expression of rRNA transcripts are related with the activation of silenced 45S rRNA genes and correlate with reduced CG and CHG methylation in the position flanking the rRNA gene transcription initiation site (44,46). Accordingly, we favor the idea that 27 S pre-rRNA over-accumulation in HSVd-infected plants can result from activation of otherwise generally repressed rRNA genes by loss in maintenance of the methylation status in both symmetric and asymmetric motifs. Additional bisulfite sequencing approaches focused on analysis of the methylation status of 5 S rDNA unities (region −143/+5) revealed that HSVd infections also induce hypomethylation in both a symmetric and asymmetric context in this rDNA family, thus reinforcing the biological relevance of this HSVd-induced phenomenon. At this point, it is important to emphasize that our data were obtained from HSVd-agroinfected plants and not from transgenic plants constitutively expressing viroid RNAs. Consequently, we cannot exclude the possibility that the analyzed tissues could represent a mix of infected and non-infected cells, thus underestimating the effects of viroid infection on host DNA methylation. Our novel and unexpected result for the HSVd pathogenesis process is consistent with that previously reported for plant–bacteria interactions*,* where 5 S rRNA repeats were demethylated at a significant level in response to infection (50). Furthermore, dynamic changes in DNA methylation patterns have been recently described during antibacterial defense in rice (51), tobacco (52) and *Arabidopsis* (23,50). Finally, in a recent study using transgenic *Nicotiana benthamiana* plants expressing the replication-associated protein (Rep) of a geminivirus, it was proposed that this type of plant DNA viruses can induce a substantial reduction in the levels of host DNA methylation at CG sites in infected plants (53). Interestingly, the geminiviruses have, as HSVd do, a nuclear replication.

Speculations on the nature of the molecular mechanism regulating this active change in rDNA methylation during HSVd infection seem premature at this stage. Intriguingly, however, a similar scenario to that observed in infected cucumber plants has been described in *Arabidopsis* when a mutant for histone deacetylase 6 (HDA6), a key regulator of gene silencing that displays a complex interrelationship with DNA methylation, was analyzed (46). Earley *et al.* reported that *hda6* mutants lose the maintenance of symmetric methylation in 45S rRNA promoter regions in parallel with a gain of *de novo* CHH methylation. Unexpectedly, and in concert with our results, increasing CHH methylation in *hda6* mutants, a typically repressive phenomenon, failed to suppress rRNA over-transcription. In addition, the siRNAs derived from the 45S IGS region hyper-accumulated in the *hda6* mutant, resembling, at least in part, that found in viroid-infected cucumber plants (Figure 2B and C). Interestingly, it was also shown that the loss of HDA6 activity induced a decrease of the symmetrical methylation of 5 S rDNA and leads to the release of 5 S rDNA silencing in mutant *Arabidopsis* plants (54). On the other hand, it was also proposed that spurious transcription of ribosomal genes by PolII can be associated with the elimination of the repressive modification regulating the rRNA expression in *Arabidopsis* (46)*.* By bearing this in mind, it is important to consider that, during infection, HSVd reprograms PolII activity to transcribe viroid RNA instead of the DNA template (55), which perhaps favors the spurious transcription of rRNAs in infected plants. Further studies are required to explore whether there is some interrelation between the observations herein and the rRNA silencing of the rRNA genes mediated by HDA6 and PolII in *Arabidopsis*. Moreover, we cannot rule out the possibility that viroid infection may induce changes in widespread dynamic DNA methylation, resembling what was observed in other pathogen–plant interactions (23,50–52). Broader analyses of DNA methylome on viroid-infected plants would help define the impact of viroid infection on DNA demethylation and host gene transcription.

In summary, the data shown here support the belief that during its pathogenesis process, HSVd induces changes in the DNA methylation of inactive rRNA genes, a previously non-described mechanism linked to viroid (or any other pathogenic RNA) infection in plants. Moreover, our findings provide new insights into aspects of the host alterations associated with the HSVd infectious cycle and constitute additional support for the emerging notion that viroid pathogenesis can be a consequence of a multilayered process involving diverse pathogen–host interactions at both the post-transcriptional and transcriptional levels.

## ACCESSION NUMBERS

NCBI/SRA accession code SRP001408.

## SUPPLEMENTARY DATA

Supplementary Data are available at NAR Online, including [56].

## FUNDING

The Spanish granting agency Direccion General de Investigacion Cientifica [BIO2011-25018 to V.P.] and from the *Prometeo* program [2011/003] from the Generalitat Valenciana. GM is the recipient of a *Marie Curie* IOF fellowship. Funding for open access charge: Direccion General de Investigacion Cientifica [BIO2011-25018].

*Conflict of interest statement*. None declared.

## Supplementary Material

Supplementary Data
