# Supplementary material for: A pathogenic non-coding RNA induces changes in dynamic DNA methylation of ribosomal RNA genes in host plants
Source: Nucleic Acids Res. 2013 Oct 30;42(3):1553–62. doi: 10.1093/nar/gkt968 (PMC3919566; doi:10.1093/nar/gkt968)
Supplement: Supplementary Data [file supp_42_3_1553__index.html]

A pathogenic non-coding RNA induces changes in dynamic DNA methylation of ribosomal RNA genes in host plants — A pathogenic non-coding RNA induces changes in dynamic DNA methylation of ribosomal RNA genes in host plants — Supplementary Data 

# A pathogenic non-coding RNA induces changes in dynamic DNA methylation of ribosomal RNA genes in host plants
